# Fitness of *Escherichia coli *strains carrying expressed and partially silent IncN and IncP1 plasmids

**DOI:** 10.1186/1471-2180-12-53

**Published:** 2012-04-04

**Authors:** Bruce Humphrey, Nicholas R Thomson, Christopher M Thomas, Karen Brooks, Mandy Sanders, Anne A Delsol, John M Roe, Peter M Bennett, Virve I Enne

**Affiliations:** 1 Bristol Centre for Antimicrobial Research, Department of Cellular and Molecular Medicine, University of Bristol, Medical Sciences Building, University Walk, Bristol, BS8 1TD, UK; 2 Pathogen Genomics, Wellcome Trust Sanger Institute, Genome Campus, Hinxton, Cambridge, CB10 1SA, UK; 3 School of Biosciences, University of Birmingham, Edgbaston, Birmingham, B15 2TT, UK; 4 Division of Animal Health and Husbandry, Department of Clinical Veterinary Science, University of Bristol, Langford, BS40 5DU, UK; 5 Centre for Immunology and Infectious Disease, Blizard Institute, Barts and The London School of Medicine and Dentistry, 4 Newark Street, London, E1 2AT, UK

## Abstract

**Background:**

Understanding the survival of resistance plasmids in the absence of selective pressure for the antibiotic resistance genes they carry is important for assessing the value of interventions to combat resistant bacteria. Here, several poorly explored questions regarding the fitness impact of IncP1 and IncN broad host range plasmids on their bacterial hosts are examined; namely, whether related plasmids have similar fitness impacts, whether this varies according to host genetic background, and what effect antimicrobial resistance gene silencing has on fitness.

**Results:**

For the IncP1 group pairwise *in vitro *growth competition demonstrated that the fitness cost of plasmid RP1 depends on the host strain. For the IncN group, plasmids R46 and N3 whose sequence is presented for the first time conferred remarkably different fitness costs despite sharing closely related backbone structures, implicating the accessory genes in fitness. Silencing of antimicrobial resistance genes was found to be beneficial for host fitness with RP1 but not for IncN plasmid pVE46.

**Conclusions:**

These findings suggest that the fitness impact of a given plasmid on its host cannot be inferred from results obtained with other host-plasmid combinations, even if these are closely related.

## Background

Antibiotic resistance is a serious threat to human and animal health and new ways to combat it are urgently needed. Broad-host range plasmids, such as those belonging to the IncN and IncP1 groups are important to the dissemination of antibiotic resistance due to their ability to replicate in a variety clinically relevant bacterial species and environments [1,2]. Indeed, both IncN and IncP1 group plasmids have been shown to encode clinically important resistance determinants such as *bla*_CTX-M_, *bla*_IMP_, *bla*_NDM_, *bla*_VIM _and *qnr *[3-8], whilst IncN plasmids have also been strongly implicated in the recent spread of *bla*_KPC _encoded carbapenemases [9].

Antimicrobial resistance can sometimes be accompanied by a reduction in biological fitness in the absence of antibiotic selection. Hence, less fit resistant bacteria may be outcompeted and displaced by fitter, susceptible bacteria in the absence of antibiotic use, leading to the suggestion that it may be possible to reduce the prevalence of antibiotic resistance by temporarily restricting prescribing. In practice, however, such approaches have enjoyed mixed success [10-14].

A fitness cost of antibiotic resistance has often been demonstrated in the case of chromosomal mutations conferring resistance, for example in the case of *fusA *mutations conferring resistance to fusidic acid [15] and *gyrA *mutations conferring resistance to fluoroquinolones [16]. However, compensatory mutations can arise at secondary sites that reduce or eliminate this cost [17]. In the case of acquired antibiotic resistance genes encoded on mobile genetic elements such as plasmids and transposons, the existence of a fitness cost is less clear. While early studies which often investigated cloning plasmids and/or laboratory strains demonstrated a cost to plasmid carriage [18-21], some more recent data using naturally-occurring plasmids and/or wild-type bacteria have failed to demonstrate significant costs and have sometimes shown a benefit. For example, the small sulphonamide and streptomycin resistance plasmid p9123 confers a 4% per generation fitness benefit in *E. coli *[22], and a benefit has also been demonstrated for some apramycin resistance plasmids isolated from bovine *E. coli *[23]. A number of antibiotic resistance encoding plasmids and transposons conferred only a low fitness cost or were cost-neutral in the wild-type *E. coli *strain 345-2RifC *in vitro *and in the pig gut [24], whilst the resistance plasmid R751 and variants of it enhanced fitness under some growth conditions in *E. coli *[25]. It is likely that the fitness cost a particular plasmid exerts on its host is variable depending on the plasmid as well as on the host itself. However, few studies have examined the fitness cost of a single plasmid on different strains of bacteria. The genetic factors, be they plasmid or host-encoded, that influence fitness are poorly understood, and it is not known whether related plasmids influence fitness in similar ways.

There are theoretically three ways in which a bacterial host can counteract the potential fitness cost exerted by antibiotic resistance genes carried on mobile genetic elements; the first is to acquire compensatory mutations, while the second is outright loss of the mobile genetic element. A third possibility is that bacteria could switch off the expression of resistance genes when they are not required whilst retaining the genes themselves in order to lower costs. We have previously demonstrated silencing of antibiotic resistance genes carried on the broad-host range plasmids pVE46 and RP1 by the wild-type *E. coli *strain 345-2RifC [26]. Following passage through the pig gut, a small proportion (0.5%) of 345-2RifC(pVE46) colonies recovered lost expression of one or more of the four resistance genes encoded on the plasmid. Such isolates had retained the pVE46 plasmid and in most cases, intact, wild-type resistance genes and promoters were present, but no resistance gene mRNA was expressed. Similar results were found for three colonies of 345-2RifC(RP1) that also lost resistance following passage through the pig gut. Antibiotic resistance gene silencing appears to be restricted to only the plasmid with minimal effect on the remainder of the genome and is thought to be due to a mutation on the chromosome of *E. coli *345-2RifC [26]. Its precise mechanism is yet to be elucidated.

Here, we examine several unexplored questions regarding the fitness impact of broad host range IncP and IncN plasmids on their hosts; namely, the effect of the host background on fitness, whether related plasmids have similar fitness impacts and the fitness impact of antimicrobial resistance gene. To facilitate this task we also report the complete nucleotide sequence of the IncN plasmid N3.

## Results and discussion

### The effect of host background on plasmid fitness impact

The effect of host genetic background on the fitness impact of plasmid RP1 in the laboratory was investigated (Table 1). Five unrelated host strains representing all four *E. coli *phylogenetic groups were studied; *E. coli *345-2RifC (group B1) and 343-9 (group D) of porcine origin, 99-24 (group D) and 99-40 (group B2) of human clinical origin (urine) and K12 (group A) JM109, a laboratory strain. Phylogenetic group B2, and to a lesser extent phylogenetic group D tend to be associated with extra-intestinal infections, whereas strains belonging to groups A and B1 are often commensals [27]. There was considerable variation in the results obtained from different host backgrounds. The fitness impacts of RP1 on the strains of animal origin (343-9 and 345-8) were significantly lower than the costs imposed on those of human origin (JM109, 99-24 and 99-40) (*p *< 0.002 in all cases).

**Table 1:** *In vitro *fitness impact of plasmid RP1 on different *E. coli *host strains

*E. coli *Host Strain	Fitness impact per generation (%)
345-2RifC	-3.3 ± 0.9

343-9	+0.8 ± 0.9

99-24	- 9.1 ± 4.2

99-40	-9.7 ± 1.4

K12 JM109	-5.8 ± 1.0

These results suggest that the fitness impact a particular antibiotic resistance plasmid confers on a given bacterial species is dependent on the genotype of the specific host strain that it is in. This conclusion is perhaps intuitive, but has to the best of our knowledge not been demonstrated for antibiotic resistance-encoding plasmids. One might expect this to be the case based on previous work by Dahlberg and Chao, who showed that amelioration of fitness costs conferred by the plasmids R1 and RP4 (very similar to plasmid RP1 used here) on *E. coli *K12 J53 depended on genetic changes in the host chromosome, thus implying a host genome component is involved in determining plasmid-encoded fitness cost [19]. Similarly, the fitness cost and stability of the plasmid pB10 was highly variable in strains of different species [28,29]. Previous studies have also shown that target mutations leading to antibiotic resistance, for example *gyrA *mutations in *Campylobacter jejuni *or 23S rRNA mutations leading to clarithromycin resistance in *Helicobacter pylori *have different fitness effects in different host backgrounds [30,31]. It is not currently known which host genetic components may be important for determining the effect a plasmid will have on host fitness and it is likely that these will vary depending on the host-plasmid combination concerned. This finding has important implications for anyone wishing to use fitness cost as a parameter to model the spread or decline of a given plasmid in a bacterial population, perhaps in response to changes in antimicrobial selection, as it highlights the need to determine fitness in several different host genetic backgrounds. Similarly, recent work has also shown that fitness cost of antimicrobial resistance is variable depending on the growth conditions used in laboratory measurements [25,32], re-iterating the need for multiple measurements to obtain accurate fitness cost estimates.

### DNA sequence analysis of N3

**Figure 1:**
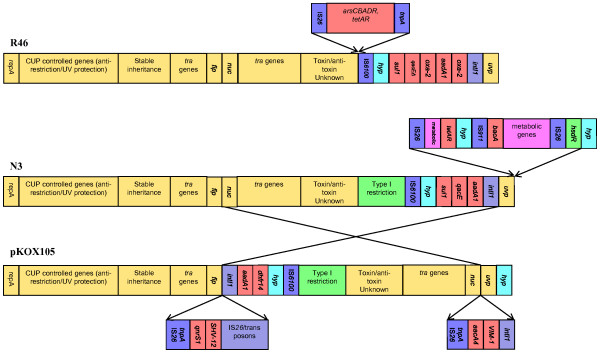
**Schematic representation of the IncN plasmids R46, N3 and pKOX105**. Not to scale. Boxes represent individual genes or groups of genes, described by name or function of the respective gene products. Blocks marked with arrows represent genes unique to each. Crossed over section between N3 and pKOX105 indicates inverted region. Colour scheme: Yellow- core IncN plasmid function, Green - Type 1 restriction system, Red - antibiotic and heavy metal resistance, Blue - mobile elements, Turquoise - hypothetical genes, Pink - Putative metabolic genes.

Despite being a well-studied archetypal plasmid isolated in the 1960s, the DNA sequence of the IncN plasmid N3 has not previously been reported [33]. Sequence analysis revealed that it is 54 205 bp in length, has a GC content of 51.1% and encodes 62 putative open reading frames (Table 2). It shares a common backbone with other IncN plasmids such as R46 [34] and the recently described multiple antibiotic resistance plasmid pKOX105 [3] (Figure 1). The shared region comprises the plasmid's replication and transfer functions as well as genes encoding stable inheritance, anti-restriction and UV protection functions. N3 also encodes a class 1 integron and, in common with pKOX105 but lacking from R46, a type 1 restriction modification system. This characteristic and the high sequence identity shown between a number of proteins encoded by the two plasmids suggests pKOX105 may have evolved from a N3-like ancestor. N3 also encodes a unique region absent from other known IncN plasmids, bordered by IS*26 *elements. This comprises the *tet*(A) genes for tetracycline resistance, a putative *bacA-*like bacitracin resistance gene and seven novel genes. Several of the novel genes are predicted to have metabolic functions, most likely amino acid metabolism. Outside this region, the high similarity between N3 and other antibiotic resistance encoding IncN plasmids suggests that they have evolved from a common ancestor and diverged from each other relatively recently. The resistance region appears to have originated as a single class 1 integron initially carrying only an *aadA1 *cassette which has subsequently acquired further cassettes and/or insertions.

**Table 2:** Positions and putative functions of open reading frames identified in plasmid N3

Gene/orf	Position	Putative Function	Closest match (Accession)^1^	Protein identity (%)
*repA*	3-722	Initiation of plasmid replication	pKOX105 (ADH29527)	100

*ardK*	1282-1623	Regulation of *ccg *genes	pEC_L46 (ADL14210)	100

*mpr*	1638-2429	Zinc metalloproteinase	pLEW517 (YP_001096387)	100

*mucB*	2580-3845	UV protection	pKOX105	100

*mucA*	3833-4273	UV protection	pEC_L46	100

*ardB*	4688-5113	Type I antirestriction system	R46 (NP_511215)	99

*ardR*	5171-5575	Regulator of CUP controlled *ccgEII *regulon	pEC_L46	100

*ccgEIII*	5585-5824	Unknown	R46	100

*ccgAI*	7332-7511	Regulation of *ccgAII *expression	R46	100

*ccgAII*	7566-7886	Prevention of RecA overproduction	pKOX105	100

*pN3_011*	7997-8341	Unknown	R46	100

*stbC*	8523-8891	Stable plasmid inheritance	R46	100

*stbB*	8893-9609	Stable plasmid inheritance	pKOX105	100

*stbA*	9618-10037	Stable plasmid inheritance	pKOX105	100

*traK*	10528-10944	Conjugal transfer protein	pKOX105	100

*traJ*	10946-12475	Conjugal transfer protein	pKP96 (YP_002332894)	100

*traI*	12475-15717	Conjugal nickase and helicase	pKP96	100

*fipA*	15717-16343	Fertility inhibition of IncP plasmids	pKM101 (AAC63100)	100

*nuc*	16517-17050	Endonuclease	pKOX105	100

*traG*	17050-18045	Conjugal transfer protein	pKOX105	100

*traF*	18087-19247	Conjugal transfer protein	pKOX105	100

*traO*	19247-20131	Conjugal transfer protein	pEC_L46	100

*traE*	20142-20840	Conjugal transfer protein	pEC_L46	100

*traN*	20830-20967	Conjugal transfer protein	pEC_L46	100

*traD*	21059-22099	Conjugal transfer protein	pEC_L46	100

*eex*	22115-22342	Entry exclusion	pKOX105	100

*traC*	22350-23063	Conjugal transfer protein	pKOX105	100

*traB*	23081-25681	Conjugal transfer protein	pKOX105	100

*traA*	25681-25998	Conjugal transfer protein	pKOX105	100

*traM*	26048-26341	Conjugal transfer protein	pKOX105	100

*korA*	26351-26632	Unknown	R46	100

*traL*	26641-27375	Conjugal transfer protein	pKOX105	100

*korB*	27484-27789	DNA binding protein	pKOX105	99

*pN3_034*	27805-28149	Unknown	pKOX105	100

*kikA*	28146-28460	Killer protein of TrbM family	pKOX105	100

*pN3_036*	28496-28807	Unknown	R46	100

*mrr*	28863-29504	Restriction endonuclease	pKOX105	100

*pN3_038*	29509-29715	Unknown	pKP96	100

*EcoRII met*	30055-31530	Modification methylase	pKOX105	100

*EcoRII*	31564-32778	Type-2 restriction enzyme	pKOX105	100

*tnpA*	33039-33833	IS*6100 *transposase	pEK499 (YP_003108355)	100

*pN3_042*	33999-34724	Unknown	pAPEC-O1-R (YP_001481449)	100

*sul1*	34938-35777	Sulphonamide resistant dihydropteroate synthase	R46	100

*qacEΔ1*	35771-36118	Quaternary ammonium compound resistance, truncated	R46	100

*aadA2*	36282-37073	Aminoglycoside adenyltransferase	p1206 (ACC77487)	100

*intI1*	37219-38232	Class 1 integrase	pKOX105	100

*uvp1*	38625-39194	Site specific recombinase	pKOX105	100

*tnpA*	39506-40210	IS*26 *transposase	pKOX105	100

*pN3_049*	40247-40750	Putative shikimate dehydrogenase (repeat protein)	*Pantotea *sp. (YP_004116848)	59

*tet*(A)	41265-42464	Tetracycline efflux protein	pQKp331H (ABS19074)	100

*tetR*	42592-43233	Repressor protein for Tet(A)	pQKp331H	100

*pN3_052*	43438-43941	Unknown	No good match	

*pN3_053*	44147-44563	Unknown	pLVPK (NP_943518)	59

*tnp orfA*	44921-45265	IS*911 *transposase, truncated	*Shigella flexneri *2a str. 2457 T (NP_835957)	80

*pN3_055*	45468-46295	Putative bacitracin resistance protein	*Acinetobacter *sp. DR1 (YP_003733303)	62

*pN3_056*	46450-47589	Putative amino acid racemase	*Pectobacterium carotovorum *PC1 (YP_003017826)	73

*pN3_057*	47686-48597	Putative LysR-type regulator	*Shewanella halifaxensis *HAW-EB4 (YP_001674862)	56

*pN3_058*	48594-49526	Putative amino acid dehydrogenase/cyclodeaminase	*Pectobacterium carotovorum *subsp. brasiliensis PBR1692 (ZP_03825565)	72

*pN3_059*	50018-50623	Putative sodium:dicarboxylate symporter	*Burkholderia dolosa *AUO158 (ZP_04944635)	56

*tnpA*	50681-51385	IS*26 *transposase	pKOX105	100

*hsdM*	51636-53192	Type I restriction enzyme EcoprrI M protein	*Escherichia coli *B185 (ZP_06660389)	90

*pN3_062*	53656-54165	Unknown	pKOX105	90

^1 ^Where more than one protein shares the exact same identity with pN3 an example is given

### The effect of the genetic composition of the plasmid on its fitness impact

The fitness impacts of the related plasmids RP1 and pUB307 and R46 and N3 on *E. coli *345-2RifC were compared. pUB307 is a derivative of RP1 which has lost the Tn*1 *transposon. The fitness impact of the Tn*1 *transposon itself has been demonstrated to be variable depending on the insertion site, with some insertion sites conferring a fitness benefit [24]. Here, pUB307 had a small fitness cost of 1.9 ± 0.8% per generation, significantly lower than that of RP1 of -3.3 ± 0.9% per generation (students t-test *p *= 0.041). In animals, carriage of neither RPI nor pUB307 influenced the ability of *E. coli *345-2RifC to colonize the pig gut compared to the plasmid-free 345-2RifC (ANOVA F value = 0.77, *p *= 0.471).

R46 was previously determined to confer a fitness cost of - 3.3 ± 1.7% per generation [24] in the laboratory, whilst no significant fitness cost in pigs was detected. In contrast, here, N3 was demonstrated to have a significantly higher fitness cost in the laboratory of 9.1 ± 1.8% per generation (students t-test *p *= 0.0002). In animals, 345-2RifC/N3 colonised the pig gut significantly worse than the plasmid free strain or 345-2RifC/R46 (ANOVA F value = 3.41, *p *= 0.035).

In the case of RP1 versus pUB307, these results suggest that the lower fitness cost of pUB307 compared to RP1 is related to the presence of less DNA. It is known that in single copy the Tn*1 *transposon does not itself have a detrimental effect on host fitness and can occasionally confer a benefit depending on the insertion site [24]. Therefore, it can be assumed that in this case the advantage gained by deletion of Tn*1 *is due to the presence of less DNA and a lowered burden of gene expression as the TEM beta-lactamase encoded by the transposon is normally expressed at high levels. As RP1 is present in multiple copies, the burden of gene expression will be higher on the plasmid than in the case of Tn*1 *insertion at a single chromosomal site. Possible additional epistatic fitness effects due to the insertion site of Tn*1 *in RP1 will also be absent in pUB307.

The reason(s) why N3 and R46 have markedly different fitness costs is less clear, as the two plasmids are a similar size and share the same replication and conjugation functions. The marked fitness difference is therefore most likely due to accessory genes. The antibiotic resistance gene complement of the two plasmids is similar, although not identical (Figure 1, Table 2). The main differences are the presence of the *arsCBADR *on R46 and a Type 1 restriction system and a number of putative metabolic genes on N3. It is likely that one or more additional genes on N3 are responsible for the high fitness cost of N3 but this hypothesis requires experimental confirmation. Alternatively, a small mutation in the core plasmid genome may also be responsible.

### The fitness impact of plasmids carrying silent antibiotic resistance genes

... In addition to variable fitness costs brought about by different host-plasmid combinations, bacteria may influence the cost of plasmid carriage by modulation of gene expression. As antibiotic resistance can impose a fitness cost on the bacterial host in the absence of antibiotic selection, one might expect phenotypic silencing of plasmid-borne antibiotic resistance genes to confer a fitness advantage. The fitness costs of the plasmids pVE46 and RP1 on *E. coli *345-2RifC had previously been established as moderate *in vitro *and non-detectable *in vivo*. Neither plasmid had a detectable cost in the pig gut [26]. However, in both cases isolates that no longer expressed the resistance genes encoded on them but retained intact and wild-type resistance genes, were recovered during the pig gut colonisation experiments [26]. Here, we investigated whether silencing of antibiotic resistance genes carried on pVE46 and RP1 had an effect on their fitness impact.

**Figure 2:**
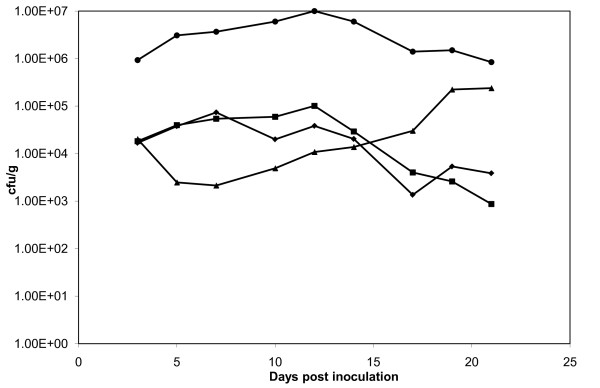
**Recovery of *E. coli *345-2RifC(pVE46) (squares), *E. coli *L5 (diamonds), *E. coli *345-2RifC(RP1) (triangles) and *E. coli *P2 (circles) from pig faeces following oral inoculation of six animals**. There was statistically no difference in recovery levels between 345-2RifC(pVE46) and L5 (ANOVA 0.5628, *p *= 0.4546). However, P2 was recovered significantly more frequently than 345-2RifC(RP1) (ANOVA 15.3169, *p *= 0.0002).

Three isolates with silent pVE46-encoded antibiotic resistance genes were investigated *in vitro*; L4, L5 and L7 (Table 3). Each isolate demonstrated variable degrees of antibiotic resistance gene silencing [26]. Pair-wise growth competition assays were performed between silent isolates and the wild-type isolates expressing all antibiotic resistance genes. Isolate L5 had a slight *in vitro *cost of -2.1% ± 1.7% per generation whilst isolates L4 and L7 had slight fitness advantages of +1.1 ± 1.4% and +1.2% ± 0.5% per generation, respectively. However, the statistical significance of these results was low and overall the impact of silencing of pVE46 genes on fitness appeared negligible. The *in vivo *ability of isolate L5 to colonize the pig gut was found to be comparable to that of 345-2RifC(pVE46) (Figure 2).

**Table 3:** Characteristics of bacterial strains and plasmids used in this study

Plasmids	Resistance Profile^1^	Resistance Genotype	Inc Group	Reference or source
pVE46	AMP, STR, SUL, TET	*bla*_OXA-2_, *sul1*, *aadA1, tet*(A)	N	[26]

R46	AMP, STR, SUL, TET	*bla*_OXA-2 _× 2, *sul1*, *aadA1, tet*(A)	N	[34]

RP1	AMP, KAN, TET	*bla*_TEM-2_, *aphA*, *tet*(A)	P	[35]

PUB307	KAN, TET	*aphA*, *tet*(A)	P	[36]

N3	STR, SUL, TET	*sul1*, *aadA1, tet*(A)	N	[33]

Bacterial Strains			Phylogenetic Group	

345-2RifC	RIF	RpoB H526Y	B1	[24]

343-9		NA	D	[24]

99-24		NA	D	[11]

99-40		NA	B2	[11]

K12 JM109	NAL	NA	A	Promega, Southampton, UK

L5^2^	RIF	*bla*_OXA-2_, *sul1*, *aadA1, tet*(A)	B1	[26]

L4^2^	RIF, TET	*bla*_OXA-2_, *sul1*, *aadA1, tet*(A)	B1	[26]

L7^2^	AMP, RIF, SUL	*bla*_OXA-2_, *sul1*, *aadA1*	B1	[26]

P1^3^	KAN, RIF	*bla*_TEM-2_	B1	[26]

P2^3^	RIF	*bla*_TEM-2_, *aphA*, *tet*(A)	B1	[26]

^1^AMP, ampicillin; KAN, kanamycin; NAL, nalidixic acid; RIF, rifampicin; STR, streptomycin; SUL, sulfamethoxazole; TET, tetracycline; NA, not applicable

^2^345-2RifC strain with pVE46 encoding silent antimicrobial resistance genes

^3^345-2RifC strain with RP1 encoding silent antimicrobial resistance genes

In contrast, antibiotic resistance gene silencing had a significant effect on the fitness of *E. coli *345-2RifC(RP1). The silent isolates P1 and P2 (Table 3) both had fitness advantages of +2.5 ± 0.5% and +4.1 ± 3.7% *in vitro*, respectively. P2 was also able to colonize the pig gut better than 345-2RifC(RP1) (Figure 2).

Surprisingly, antibiotic resistance gene silencing did not confer a fitness advantage on isolates carrying the pVE46 plasmid, *in vivo *or *in vitro*. This suggests that in this case antibiotic resistance gene silencing may have occurred by random chance that was fortuitously detected, or that if it exists, any fitness advantage only manifests itself under conditions not measured by our current assays. This observation may be explained by the fact that the initial cost conferred by carriage of pVE46 on *E. coli *345-2RifC was moderate, 2.8 ± 0.9%, per generation. However, previous studies did show that pVE46-encoded antibiotic resistance genes were able to revert back to resistance at rates varying between 10^-6 ^and 10^-10 ^i*n vitro *[26] suggesting that such strains may still pose a clinical threat.

In contrast, silencing of antibiotic resistance genes encoded on the plasmid RP1 conferred a significant fitness benefit both *in vivo *and *in vitro*. Such a strategy could be deemed beneficial for the bacterium, particularly if they were able to revert to antibiotic resistance again when challenged with antibiotic. However, this was not the case as none of the isolates with silent RP1 antibiotic resistance genes (P1, P2 or P3) were able to revert back to resistance in the laboratory. This suggests that the genetic event responsible for antibiotic resistance gene silencing of RP1 is not readily reversible, for example a transposon insertion or DNA deletion. Under such conditions one would expect the silenced DNA to eventually be lost, but until then it may act as an environmental reservoir of resistance genes.

In theory any fitness effects observed in silent isolates could also be attributed to unrelated mutations that may have arisen in the pig gut prior to their isolation. However, the silent isolate L5 is not known to carry any mutations compared to the wild-type 345-2RifC(pVE46) strain, whilst the possible role of unrelated mutations in the remaining isolates is yet to be determined (B.H. V.I.E and N.R.T, unpublished data).

## Conclusions

Overall, the results presented here show that the fitness balance between the host genotype and a given resistance plasmid is extremely delicate and that even minor differences in the host or in the plasmid can have substantial effects on fitness. Future studies on the subject should therefore investigate multiple hosts in order to draw any general conclusions about a particular plasmid. Without better molecular understanding of the processes involved, it is difficult to predict the fitness impact of a given host-plasmid association, and hence difficult to make predictions about the spread or decline of associated antibiotic resistance phenotypes. It is therefore important to study molecular host-plasmid interactions. In the absence of such data one should preferably use a range of host strains and plasmids when studying the fitness of a particular resistance phenotype. As plasmids belonging to the IncN and IncP1 groups are broad-host range and conjugative they will likely move from host to host until they encounter one where costs are negligible and subsequently go on to thrive with that host. Thus, such plasmids may be of particular concern in the dissemination of novel antibiotic resistance phenotypes.

In addition, bacteria can sometimes "hide" their resistance genotype by silencing it. This can create a fitness advantage for the bacteria, whereas in other cases the silent phenotype is reversible, indicating that there is a risk of treatment failure in anyone infected by bacteria with silent genes and being treated by one of the affected antibiotics.

Based on these observations, further work should now concentrate on understanding the molecular mechanisms responsible so that the underlying process are understood and used to help develop better treatment and prevention and control strategies.

## Methods

### Bacterial strains and plasmids

*E. coli *345-2RifC, *E. coli *345-8 and 343-9 are all commensal isolates of porcine origin. *E. coli *345-2RifC is marked with a no-cost rifampicin-resistance mutation in RpoB (H526Y). Strains 99-24 and 99-40 are human urinary isolates, whilst *E. coli *K12 JM109 is a laboratory strain. Study strains were chosen on the basis that they did not carry acquired antibiotic resistance genes and that they exhibited good growth characteristics in laboratory media, with doubling ranging between 21 and 27 minutes in nutrient broth. Their phylogenetic group was determined as described previously [27]. The relatedness of the isolates was investigated by randomly amplified polymorphic DNA (RAPD) PCR [37].

The broad-host range plasmids RP1, pUB307, R46, pVE46 and N3 were introduced into host strains by conjugation using the agar mating method [26]. The 345-2RifC(pVE46) strain used was a variant passaged in the laboratory, the same from which silent isolates arose [26]. Derivatives of 345-2RifC(pVE46) and 345-2RifC(RP1), carrying silent antibiotic resistance genes were as described previously [26]. The characteristics of strains and plasmids used in this study are listed in Table 3.

### DNA sequencing and analysis

DNA of IncN plasmid N3 was prepared by alkaline SDS maxiprep and CsCl/EtBr density gradient centrifugation [38]. The *E. coli *N3 plasmid was sequenced to approximately 37-fold shotgun sequence, totalling 1711 end sequences, from pUC19 (with insert sizes of 2-4 kb; 4-6 kb) genomic shotgun libraries that were sequenced using big-dye terminator chemistry on ABI3730 automated sequencers. The assembly was generated using phrap2gap. All repeat regions and gaps were bridged by read-pairs or end-sequenced polymerase chain reaction (PCR) products again sequenced with big dye terminator chemistry on ABI3730 capillary sequencers. The sequence was manipulated to the 'Finished' standard [39].

### Competition experiments to assay *in vitro *fitness

To assess the fitness impact of the plasmids upon *E. coli *host strains growth competition between plasmid-carrying and plasmid-free isogenic strain pairs was carried out as described previously in Davis minimal medium with 25 mg/ml glucose (DM25) [24]. To estimate bacterial counts, competition cultures were diluted as appropriate and spread in triplicate onto IsoSensitest agar (Oxoid) and onto IsoSensitest agar containing the relevant antibiotic. For the competition between the silent strains L5 or L7 and 345-2RifC(pVE46) the agar contained tetracycline at 25 μg/ml, and for L4 it contained streptomycin at 25 μg/ml. For competition between 345-2RifC(RP1) and P1 or P2 agar contained ampicillin at 25 μg/ml. For competition between wild-type plasmids and their respective host strains it contained ampicillin for RP1 carrying strains, and tetracycline for the pUB307 and N3 carrying strains. Six replicates of each competition experiment were performed. Average per generation fitness (W) was calculated as W = 1 - b, where b is equal to t he gradient of the graph of ln(strain x count/strain y count) per transfer, divided by the number of generations per transfer (T). T was calculated as ln(dilution factor)/ln(2). The students t-test was used to estimate the statistical significance of results.

### Investigation of *in vitro *reversion to resistance

The recovery of resistance by isolates with intact but silent RP1 encoded resistance genes was investigated by spreading undiluted and serially diluted overnight nutrient broth cultures onto IsoSensitest agar containing the appropriate antibiotic (ampicillin, 25 μg/ml; kanamycin 30 μg/ml; tetracycline, 25 μg/ml). To calculate reversion frequencies, total cell counts were obtained following plating serial dilutions of the same culture onto antibiotic-free medium.

### Animal experiments

Animal experiments were carried out using a modified method of that described previously [24]. For each experiment, six organic piglets from two litters of Saddleback-Duroc cross, weaned at five weeks of age, were housed as a single group for two weeks, to allow the animals to acclimatize to their surroundings. They were then randomly separated into two groups of three into pens with individual HEPA filtration and fed a standard organic feed (Organic feed company, grower/finisher pellets, UK) ad libitum. All procedures complied with the Animals (Scientific Procedures) Act 1986 and were performed under Home Office License.

Briefly, bacterial strains (*E. coli *345-2RifC(pVE46), 345-2RifC(RP1), L5 and P1) were inoculated separately into six piglets as a single dose of 10^10 ^cfu per animal by oral gavage. Faecal samples were collected from each animal by digital manipulation on day 3, 5, 7, 10, 12, 14, 17, 19 and 21 post-inoculation and analysed within 24 hours. One gram of faeces was suspended in nine millilitres of saline and plated at appropriate dilutions onto six MacConkey agar plates containing 50 μg/ml rifampicin (detection limit 2 cfu/g). They were incubated overnight at 37°C and colonies obtained replica plated onto MacConkey agar containing 50 μg/ml rifampicin with ampicillin (25 μg/ml), tetracycline (25 μg/ml), sulfamethoxazole (500 μg/ml) or streptomycin (25 μg/ml) for L5, and rifampicin with ampicillin, tetracycline or kanamycin (30 μg/ml) for P1, followed by replica plating onto MacConkey agar with rifampicin only.

### Nucleotide sequence accession number

The N3 DNA sequence has been submitted to EMBL under the accession number FR850039.

## Competing interests

The authors declare that they have no competing interests.

## Authors' contributions

BH, KB, MS, NRT and VIE performed the experimental work and data analysis. AAD and PMB participated in the study design. NRT, CMT, JMR and VIE co-ordinated the study and participated in the design. BH, NRT, CMT and VIE drafted the manuscript. VIE and PMB conceived the study. All authors read and approved the final manuscript.
